# Predictors of Post-Operative Hospital Length of Stay Following Complete Repair of Tetralogy of Fallot in a Pediatric Cohort in the North of England

**DOI:** 10.1007/s00246-023-03287-7

**Published:** 2023-09-12

**Authors:** Adenike M. Adesanya, Kate E. Best, Louise Coats, Judith Rankin

**Affiliations:** 1 Population Health Sciences Institute, Faculty of Medical Sciences, Newcastle University, Baddiley-Clark Building, Richardson Rd, Newcastle Upon Tyne, NE2 4AX UK; 2 Leeds Institute of Health Sciences, University of Leeds, Leeds, UK; 3 Freeman Hospital, Newcastle Upon Tyne Hospitals NHS Foundation Trust, Newcastle Upon Tyne, UK; 4 NIHR Applied Research Collaboration North East and North Cumbria, Newcastle Upon Tyne, UK

**Keywords:** Tetralogy of Fallot, Post-operative Length of Stay, North of England, Extubation, Congenital Heart Defects

## Abstract

We sought to estimate the median post-operative length of stay (PLOS) and predictors of PLOS following tetralogy of Fallot (ToF) repair at a specialist surgical center in the North of England. The local National Congenital Heart Disease Audit dataset was used to identify patients aged < 2 years who underwent surgical repair for ToF between 1 January 1986 and 13 May 2022. Coefficients representing the median change in PLOS (days) according to predictors were estimated using Quantile regression. There were 224 patients (59.4% male, median age = 9 months, interquartile range (IQR) 5–13 months) with a median PLOS of 9 days (IQR 7–13). In the univariable regression, age (months) and weight (kg) at operation (β =  − 0.17, 95% CI: − 0.33, − 0.01) and (β =  − 0.53, 95% CI: − 0.97, − 0.10), previous (cardiac or thoracic) procedure (β = 5, 95% CI:2.38, 7.62), procedure urgency (elective vs urgent) (β = 2.8, 95% CI:0.39, 5.21), bypass time (mins) (β = 0.03, 95% CI:0.01, 0.05), cross-clamp time (mins) (β = 0.03, 95% CI:0.01, 0.06) and duration of post-operative intubation (days) (β = 0.81, 95% CI:0.67, 0.96), were significantly associated with PLOS. Previous procedure and intubation time remained significant in multivariable analyses. Some patient and operative factors can predict PLOS following complete ToF repair. Information on PLOS is important for health professionals to support parents in preparing for their child's discharge and to make any necessary practical arrangements. Health commissioners can draw on evidence-based guidance for resource planning. The small sample size may have reduced the power to detect small effect sizes, but this regional study serves as a foundation for a larger national study.

**Supplementary Information:**

The online version contains supplementary material available at 10.1007/s00246-023-03287-7.

## Clinical Perspective

### What is New?

- In a retrospective pediatric cohort study set in the North of England, we identified a median post-operative length of stay of 9 days and the associated predictors following complete surgical repair for tetralogy of Fallot.
- Increasing age and weight at operation were associated with decreasing length of stay in hospital.
- A significant previous cardiac or thoracic procedure, urgent procedure, increasing total bypass time, total cross-clamp time and duration of post-operative intubation, were associated with increasing length of stay in hospital.

### What are the Clinical Implications?

- Several patient and operative factors, some of which have implications for management strategies, are associated with a longer or shorter stay in hospital after surgical repair for tetralogy of Fallot.
- The increasing evidence for the safety of early extubation following congenital cardiac surgery suggests early extubation may be a clinical practice to consider implementing in the North of England.

## Introduction

In the 2020/21 financial year, there were approximately 7000 pediatric procedures performed to repair or palliate children with congenital heart disease (CHD) aged 16 years and under in the United Kingdom (UK) [1]. The numbers have risen steadily over time, although there was a significant decrease in activity due to the impact of the COVID-19 pandemic [1]. In Newcastle upon Tyne, which hosts a Level-1 (surgical) CHD center in the North of England, 1121 pediatric CHD repairs and palliations were performed between 2019 and 2022 [2]. This figure amounts to 6% of all the pediatric procedures performed in the UK for CHD.

UK post-operative mortality rates are easily accessible from the National Institute for Cardiovascular Outcomes Research (NICOR) website [3]. Information regarding the post-operative length of stay (PLOS) is very limited and, where available, there are some inconsistencies in the way hospital length of stay is reported. A national UK study reported a median PLOS of 7 days following any cardiac operation in all CHD patients but did not provide CHD subtype specific estimates [4]. Another European population-based study including registry data from the UK reported the median total length of stay in hospital of 24.4 days in the first year of life for children with a CHD [5].

In the United States (US), the Society of Thoracic Surgeons report the median procedure-specific PLOS for CHD correction or palliation performed between 2017 and 2012, with PLOS ranging from 11 to 56 days following repair of ventricular septal defect and the Norwood procedure, respectively [6]. Studies from the US show that PLOS is significantly associated with CHD severity, distance from home to hospital, previous surgeries, morbidities (extra-cardiac anomalies, cyanosis, oxygen requirement), post-operative complications (heart block, diaphragm paralysis, organ-system failure, necrotizing enterocolitis, unplanned cardiac catheterisation) and surgical factors (time on bypass) [7–9]. It is not clear whether the findings from these US studies are applicable to UK populations.

Tetralogy of Fallot (ToF) is one of the most common complex, cyanotic CHD with significant improvement over time in the outcomes of surgical intervention [10]. NICOR specific procedure summary statistics, report a near 100% 30-day survival rate for ToF repair procedures in the UK [11].

Information on PLOS is important for parents, commissioners and health professionals. When CHD is diagnosed antenatally, procedure-specific estimates of PLOS can be used by parents in conjunction with mortality rates, to aid decision-making in terms of pregnancy continuation. Postnatally, estimates of PLOS allow parents to make practical arrangements (e.g., leave from work, childcare for siblings) and can better prepare them for their child’s discharge date [12]. The NHS devotes just 1% of its specialized commissioning budget to CHD services [13], which must cover hospital stays costing approximately £1,224, £840 or £433 per day for intensive, high dependency care and special care, respectively [14]. Therefore, information on PLOS can help commissioners ensure adequate resources (e.g., bed spaces, special care nurses) are available for children recovering from CHD repair. Identification of predictors of PLOS can lay the foundations for the development and validation of clinical prediction models aimed to predict PLOS.

The aim of this study was to estimate the median and range of PLOS, and to identify predictors of PLOS following ToF repair in children presenting to a level 1 specialist surgical center for CHD in the North East of England.

## Method

### Data Source

Newcastle upon Tyne Hospitals NHS Trust (NUTH) forms part of the national CHD service commissioned through NHS England Specialised Services to deliver care to both adults and children as a level 1 specialist surgical center. NUTH also hosts the North East and North Cumbria Congenital Heart Disease (NENC-CHD) Network which covers a population of 3.2 million people in the North East and North Cumbria [15]. The center which regularly submits data on surgical outcomes to NICOR has been collecting data since 1986, with a most recent data quality indicator score of 99.5% in 2021/22 [15].

Patients who underwent surgical repair for ToF in a single operation between 1 January 1986 and 13 May 2022 (data extraction date), whether or not it was preceded by any previous procedure, were identified from the local National Congenital Heart Disease Dataset Audit (NCHDA). As defined by the NCHDA Version 6.1, ToF repair primary procedure was indicated as: (1) 120,511. Atrioventricular septal defect (AVSD) & ToF repair; (2) 122,601. ToF repair; (3) 122,613. ToF repair with transannular patch; (4) 122,620. ToF repair without transannular patch. We excluded patients who had a diagnosis of absent pulmonary valve syndrome, pulmonary atresia, ventricular septal defect and major aortopulmonary collateral arteries (MAPCAS), double outlet right ventricle, unbalanced ventricles or isomerism.

Age at operation was calculated prior to the secure transfer of data to the researchers. PLOS (days) was calculated using date of ToF repair procedure and date of discharge recorded. Patients aged over 2 years at the time of operation (n = 36), with missing date of discharge (n < 5), were excluded. We included patients who died prior to discharge (n < 5), as their hospital length of stay was relatively short. We have set the ethnicity subgroup into two groups consisting of white and ethnic minorities (excluding white minorities) to avoid identifying patients in ethnic groups having less than three individuals.

### Patient Public Involvement

We developed a patient public involvement questionnaire (supplementary material 1) to understand what patients and their carers felt they might like to know from this study about their length of stay in hospital. We received responses which highlighted the importance of receiving clear information on the expected length of stay or recovery time in hospital for calming the fears and anxieties about what to expect, organizing life administration such as time off work, travel to and from hospital. The responses also highlighted a need to know if the distance from place of residence to the hospital would impact the care they received or how long they had to spend in hospital after an operation.

### Statistical Analysis

Descriptive statistics were calculated as frequency counts and percentages for categorical variables and median with interquartile range (IQR) for continuous variables. Univariable quantile regression was performed to examine the association between median PLOS and the following predictors:

- Antenatal diagnosis;
- Age at procedure (months);
- Ethnicity;
- Sex;
- Presence of a comorbidity (as defined by the NCHDA Version 6.1);
- Weight at procedure (kg);
- Procedure urgency (elective vs urgent);
- Significant previous cardiac or thoracic procedure as a measure of severity;
- Sternotomy sequence;
- Total bypass time (minutes);
- Total cross-clamp time (minutes);
- Duration of post-operative intubation (days);
- Year of procedure;
- Index of multiple deprivation quintile (IMD) derived from the postcode of place of residence as a measure of socioeconomic status [16]; and
- Shortest distance between postcode of place of residence and the hospital (miles) on Google Maps.

Where least squares regression estimates the conditional mean, quantile regression estimates the conditional median (or other selected quantile) and is appropriate for modeling non-normally distributed outcomes. The coefficient estimated in the median quantile regression for a continuous predictor can be interpreted as the median difference in PLOS (days) for a unit increase in the predictor. For a categorical predictor, the coefficient is interpreted as the median difference in PLOS in the category of interest vs the reference category. For each variable of interest, a different multivariable quantile regression model was fitted, adjusting only for the minimal sufficient adjustment set (i.e., the smallest selection of available variables required to reduce confounding). Daggity was used to identify the adjustment set for estimating the total effect of the predictor in question on PLOS [17]. The adjustment set for each multivariable model is shown in Table 1.

**Table 1:** Directed acyclic graph adjustment set

Variable of interest	Minimal sufficient adjustment set for total effect
Comorbidity	Ethnicity, sex
IMD quintile	Year of procedure
Sex	Ethnicity, year of procedure
Ethnicity	IMD quintile, year of procedure
Previous procedure	Comorbidity present, ethnicity, sex, IMD quintile, year of procedure
Sternotomy sequence	Comorbidity present, distance, ethnicity, sex, IMD quintile, antenatal diagnosis, previous procedure (measure of severity), year of procedure
Weight at operation	Age at operation, comorbidity present, distance, ethnicity, sex, IMD quintile, antenatal diagnosis, previous procedure (measure of severity), sternotomy sequence, year of procedure
Antenatal diagnosis	Comorbidity present, ethnicity, sex, IMD quintile, previous procedure (measure of severity), year of procedure
Age at operation	Comorbidity present, distance, ethnicity, sex, IMD quintile, antenatal diagnosis, previous procedure (measure of severity), sternotomy sequence, year of procedure
Total bypass time	None
Total cross-clamp time	Age at operation, total bypass time, comorbidity present, distance, ethnicity, sex, IMD quintile, antenatal diagnosis, previous procedure (measure of severity), year of procedure, weight at operation
Duration of post-operative intubation	Age at operation, total bypass time, comorbidity present, total cross-clamp time, ethnicity, sex, IMD quintile, antenatal diagnosis, previous procedure (measure of severity), year of procedure, weight at operation
Year of procedure	None
Distance to hospital	Comorbidity present, ethnicity, IMD quintile, antenatal diagnosis, previous procedure (measure of severity), year of procedure

Multiple imputation with chained equations was performed with 20 imputations to estimate missing values for antenatal diagnosis, ethnicity, presence of a comorbidity, weight at procedure, procedure urgency, total bypass and cross-clamp time and duration of post-operative intubation. PLOS and all of the predictors included in the models were included in the multiple imputation. Statistical analysis was performed in Stata 17, with p < 0.05 used as the nominal significance level.

## Results

There were 224 patients aged 2 years and under who underwent an operation for ToF complete repair at Freeman Hospital between 1 January 1986 and 13 May 2022. Fifty-nine percent were male and 87.5% were white. A summary of the demographic, clinical, surgical, and outcome characteristics are shown in Table 2.

**Table 2:** Summary of demographic, clinical, surgical, and outcome characteristics of the study cohort

Predictor	N (%) or median (IQR)	N out of 224 (% missing)
Antenatal diagnosis		29 (12.9)
Yes	65 (29)	
No	130 (58)	
Age at operation (months)	9.4 (5.3—13.2)	
Ethnicity		11 (4.9)
Ethnic minorities (excluding white minorities)	17 (7.6)	
White	196 (87.5)	
Sex		
Male	133 (59.4)	
Female	91 (40.6)	
Comorbidity present		16 (7.1)
Yes	119 (53.1)	
No	89 (39.7)	
Weight (kg)	7.8 (6.3—9.0)	3 (1.3)
Previous procedures		
No significant cardiac or thoracic procedure	202 (90.2)	
Shunt, stent, valvotomy or other significant thoracic surgery	22 (9.8)	
Procedure urgency		50 (22.3)
Elective	131 (58.5)	
Urgent	43 (19.2)	
Sternotomy sequence		
First sternotomy	209 (93.3)	
Second or third sternotomy	15 (6.7)	
Total bypass time (mins)	122 (100–140)	36 (16.1)
Total bypass cross-clamp time (mins)	71 (56–91)	36 (16.1)
Duration of post-operative intubation (days)	3 (2–6)	42 (18.8)
Post-operative length of stay (days)	9 (7–13)	
Year of Procedure	2010 (2005–2016)	
IMD Quintile	2 (1–3)	
Distance to hospital (miles)	20.0 (7.9–42.9)	

Here we present the median and IQR for: age at operation (median 9 months; IQR 5 – 13 months), PLOS (median 9 days; IQR 7 – 13 days); total bypass time (median 122 min; IQR 100 – 140 min), and total bypass cross-clamp time (median 71 min; IQR 56 – 90 min). The median duration of post-operative intubation was 3 days (IQR 2 – 6 days). The median distance from postcode of place of residence to hospital was 20.0 miles (IQR 7.9 – 42.9 miles).

A significant previous cardiac or thoracic procedure was performed in 9.8% of patients. The significant previous procedures included: stent placement in right ventricular outflow tract, modified R Blalock interposition shunt, modified L Blalock interposition shunt, systemic-to-pulmonary arterial shunt procedure, procedure involving constructed cardiac conduit/shunt, balloon pulmonary valvotomy, tracheo-oesophageal fistula repair.

In the univariable regression (Table 3), for each month increase in age at operation, the median PLOS decreased by 0.17 days (95% CI: − 0.33, − 0.01), and for each kg increase in weight at operation, the median PLOS decreased by 0.53 days (95% CI: − 0.97, − 0.10).Significant previous cardiac or thoracic procedure (β = 5, 95% CI: 2.38, 7.62), procedure urgency (elective vs urgent) (β = 2.8, 95% CI: 0.39, 5.21), total bypass time (mins) (β = 0.03, 95% CI: 0.01, 0.05), cross-clamp time (mins) (β = 0.3, 95% CI: 0.01, 0.06) and duration of post-operative intubation (days) (β = 0.81, 95% CI: 0.67, 0.96) were associated with increased PLOS in univariable models. There were no significant univariable associations with year of procedure, ethnicity, sex, comorbidities, distance between postcode of place of residence and the hospital or IMD quintile as a measure of socioeconomic status.

**Table 3:** Quantile regression models of post-operative length of stay in the study cohort

Predictor	Univariable	*p*-value	Multivariable	*p*-value
	Coefficient (95% CI)		Coefficient (95% CI)	
Antenatal diagnosis				
Yes	1.75 (− 0.13, 3.63)	0.07	0.71 (− 1.68, 3.10)	0.56
No	(reference category)		(reference category)	
Age at operation (months)	− 0.17 (− 0.33, − 0.01)	0.04	− 0.18 (− 0.41, 0.05)	0.12
Ethnicity				
Ethnic minorities (excluding white minorities)	1.85 (− 1.24, 4.94)	0.24	1.36 (− 1.67, 4.35)	0.37
White	(reference category)		(reference category)	
Sex				
Female	1 (− 0.68, 2.68)	0.24	0.59 (− 1.09, 2.26)	0.49
Male	(reference category)		(reference category)	
Comorbidity present				
No	− 1 (− 2.59, 0.59)	0.22	− 1 (− 2.62, 0.62)	0.23
Yes	(reference category)		(reference category)	
Weight at operation (kg)	− 0.53 (− 0.97, − 0.1)	0.02	− 0.31 (− 1.04, 0.42)	0.40
Previous procedure				
Shunt, stent, valvotomy or other significant thoracic surgery	5.0 (2.38, 7.62)	< 0.001	4.75 (1.66, 7.85)	0.003
No significant cardiac or thoracic procedure	(reference category)		(reference category)	
Procedure urgency				
Urgent	2.8 (0.39, 5.21)	0.02	1.9 (− 0.73, 4.53)	0.16
Elective	(reference category)		(reference category)	
Sternotomy sequence				
Second or third sternotomy	2 (− 1.68, 5.68)	0.29	0.75 (− 3.61, 5.11)	0.74
First sternotomy	(reference category)		(reference category)	
Total bypass time (mins)	0.03 (0.01, 0.05)	0.002	No DAG	No DAG
Total bypass cross-clamp time (mins)	0.03 (0.01, 0.06)	0.02	− 0.01 (− 0.07, 0.05)	0.79
Duration of post-operative intubation (days)	0.81 (0.67, 0.96)	< 0.001	0.76 (0.56, 0.95)	< 0.001
IMD Quintile				
2	− 1 (− 3.05, 1.05)	0.34	− 0.59 (− 2.62, 1.44)	0.57
3	− 1 (− 3.76, 1.76)	0.48	− 0.59 (− 3.30, 2.12)	0.67
4	1 (− 1.55, 3.55)	0.44	1.176 (− 1.33, 3.68)	0.36
5	− 1 (− 3.76, 1.76)	0.48	− 0.53 (− 3.24, 2.18)	0.7
1	(reference category)		(reference category)	
Distance to hospital (miles)	− 0.01 (− 0.04, 0.02)	0.52	− 0.01 (− 0.04, 0.03)	0.78
Year of procedure	0.06 (− 0.06, 0.18)	0.30	−	-

In the multivariable models (Table 3), a significant previous cardiac or thoracic procedure (β = 4.75, 95% CI: 1.66, 7.85) and duration of post-operative intubation (β = 0.76, 95% CI: 0.56, 0.96) significantly predicted PLOS.

## Discussion

In this study, we have estimated the median PLOS following a complete surgical repair of ToF in a level 1 CHD surgical center in the North East of England to be 9 days. Separately, age and weight at operation, a significant previous cardiac or thoracic procedure, urgent procedure, total bypass time, total bypass cross-clamp time, and duration of post-operative intubation were associated with PLOS. In multivariable models, a significant previous cardiac or thoracic procedure and the duration of post-operative intubation were associated with PLOS.

The reported median PLOS of 9 days in our study is different to that reported by the Society of Thoracic Surgeons Congenital Heart Surgery Database in the US who have reported a median PLOS of 12.8 days in operations conducted within the four-year period from July 2017 to June 2021 [6]. However, they have not provided specific details about which age group the patients belonged to, which type of repair was performed or any other variables included, so there is some limit to how comparable the findings are. Additionally, variations may arise from differences in the healthcare system organization, with the US being insurance-based and the UK having a universal healthcare system. In the report published for the four-year period from 2005 to 2009 [18], they reported a median PLOS of 9.9 days which is more similar to our findings, but the same caveat above remains about how their patient group was defined.

Previous studies have investigated the association between PLOS and gestational age and weight at birth, however these factors may not be modifiable where early delivery is recommended [19–24]. Our findings that increasing age and weight at operation significantly predict a decrease in length of stay following surgical repair support the need for sufficiently tailored peri-operative nutritional protocols for patients with a CHD to help improve outcomes [25, 26].

The presence of a previous procedure including shunt, stent, valvotomy, or other significant thoracic surgery was used as a measure of ToF severity. It is not surprising that in both the univariable and multivariable model, a previous procedure was associated with an extra 5-day stay in hospital following complete repair for ToF. However, palliation remains preferable to early primary repair in some patients with indications such as prematurity, complex anatomy, small pulmonary artery size, and comorbidities [27].

We note that IMD and the distance between place of residence and hospital had no significant association with PLOS. Using a population level measure of deprivation may not be a true representation of individual deprivation leading to ecological fallacy, i.e., errors in drawing inferences from a group, however IMD is an important measure of the effect of socioeconomic deprivation on health outcomes.

There is increasing evidence for the safety of early extubation and its associated factors which are predictive of extubation success following congenital cardiac surgery including ToF repair [28–31]. As the duration of post-operative intubation remained a significant predictor of PLOS in multivariate models, early extubation either on the table or within a few hours following ToF surgery may significantly reduce the length of stay in hospital. A consequence of decreasing intubation time is moderating the exposure to the neurotoxic effects of anesthetic and sedative agent exposure in young children with CHDs [32].

Parental anxiety in families of children undergoing cardiac procedures for a CHD has been linked to the distance between home and the surgical center [33]. Service users from the large region which falls under the NHS England Specialised Services commissioned North East and North Cumbria CHD level 1 specialist surgical center may find some reassurance in knowing that the further the distance they live from the center does not have a significant effect on how long a patient spends in hospital following their operation.

With significant improvements in the management of ToF and low mortality rates, PLOS serves as an appropriate outcome measure. Identifying predictors of PLOS ensures adequate resources (e.g., beds, special care nurses) are available; health professionals are able to develop algorithms to minimize PLOS while maintaining health outcomes; parents can make practical arrangements around leave from work, childcare for siblings, etc., and in complex CHD cases, can be incorporated into the information provided to parents to support decision-making around pregnancy continuation.

This study has several strengths. The data underlying this study have achieved high data quality indicator scores and the data quality was checked for completeness by a data manager from the surgical center. However, it also has some limitations. The small sample size of 224 patients means we are unable to rule out type II errors where we found non-significant associations, particularly in the multivariable analyses, where the effect size was relatively small or where the categorical variables were unbalanced (e.g., low prevalence of specific categories). While we were able to account for missing data via multiple imputation, the small sample size may have reduced the power to detect small effect sizes.

As very few patients in this study died in hospital (*n* < 5), and their hospital length of stay was relatively short, they were included in the analysis. However, this undoubtedly impacts the results. Alternative approaches could be to restrict the analysis to those patients who lived or to assign those who die the "worst" outcome [34]. In truth, however, death is a competing risk and should be considered as such when investigating time-related outcomes.

We also were not adequately powered to examine interactions between variables; for example, it may have been interesting to look at interaction between age and weight, perhaps those children with low weight for age fare worse than low weight due to being younger. As this is regional data derived from one surgical center, we are unable to account for variation in practice across different units in the UK, which may limit how applicable the findings are outside of the region.

This regional study serves as a foundation for a larger UK national study and an exemplar for investigating length of stay in hospital for children with other CHD subtypes.

### Supplementary Information

Below is the link to the electronic supplementary material.

Supplementary file1 (DOCX 16 KB)
