# Supplementary material for: Predictors of Post-Operative Hospital Length of Stay Following Complete Repair of Tetralogy of Fallot in a Pediatric Cohort in the North of England
Source: Pediatr Cardiol. 2023 Sep 12;45(1):92–9. doi: 10.1007/s00246-023-03287-7 (PMC10776676; doi:10.1007/s00246-023-03287-7)
Supplement: Supplementary file 1 — Supplementary file1 (DOCX 16 KB) [file 246_2023_3287_MOESM1_ESM.docx]

**Supplementary Material 1. Patient Public Involvement Questionnaire**

**Hospital Length of Stay Project**

**Project Ref:**

**PI Professor Judith Rankin**

**Patient Public Involvement Questionnaire**

We are doing a project on how long children and adults with congenital heart disease spend in hospital. We will be using nationally collected data to look into this.

We would like to understand what things you feel are important to know about for example is it just the length of stay that matters to you, how often you have to stay in hospital or which hospital you stayed in. There may be other things it is important for us to consider

Please let us know what you think about this area of research and we use your views to help us design what we do. You can write any thoughts or ideas below:

This research is funded by the Newcastle Upon Tyne Hospitals NHS Charity.

If you would like to know more about the research or have any questions.

Please contact judith.rankin@newcastle.ac.uk
